# Tackling Health Disparities for People Who Are Homeless? Start with Social Determinants

**DOI:** 10.3390/ijerph14121535

**Published:** 2017-12-08

**Authors:** Amanda Stafford, Lisa Wood

**Affiliations:** 1 Royal Perth Hospital, Perth, WA 6000, Australia; 2 School of Population and Global Health and Centre for Social Impact, The University of Western Australia, Crawley, WA 6009, Australia; Lisa.Wood@uwa.edu.au

**Keywords:** homeless, social determinants of health, health inequalities, health sector

## Abstract

Background: Homelessness is associated with enormous health inequalities, including shorter life expectancy, higher morbidity and greater usage of acute hospital services. Viewed through the lens of social determinants, homelessness is a key driver of poor health, but homelessness itself results from accumulated adverse social and economic conditions. Indeed, in people who are homeless, the social determinants of homelessness and health inequities are often intertwined, and long term homelessness further exacerbates poor health. Aggregated health service data can mask this, and case histories thus provide important insights. Methods: This paper presents three case histories of homeless patients seen at an inner city public hospital in Perth, Western Australia. The case histories draw on several data sources: hospital data, information collected from rough sleepers and clinical observations. Estimates of the cost to the health system of the observed hospital usage by the three patients are included. Findings: The case histories illustrate the interplay of social determinants of health in homelessness that help explain the high level of hospital usage by rough sleepers. The cumulative healthcare costs for the three individuals over a 33 months period were substantial. Hospital attendance plummeted even in the short term when housing needs were addressed. Conclusions: Treating homelessness as a combined health and social issue is critical to improving the abysmal health outcomes of people experiencing homelessness. In addition, the enormous economic costs of hospital care for people who are homeless can be reduced when housing and other social determinants are taken into account.

## 1. Introduction

Around the world, enormous health inequalities are found amongst people experiencing homelessness. Being homeless is associated with shorter life expectancy [1], higher morbidity and greater usage of acute hospital services [2]. People experiencing homelessness are also less likely to access primary and preventive health services [3] resulting in increased risk for later-stage diagnosis of disease [4], poor control of manageable conditions (e.g., hypertension, diabetes) and hospitalization for preventable conditions (e.g., skin or respiratory conditions). A 2016 Perth survey of 307 people sleeping rough found that 71% had presented at an Emergency Department (ED) in the previous six months, many on multiple occasions [5]. Data from UK, US and Canada similarly reports a high prevalence of homeless individuals in ED presentations and unplanned hospital admissions [6,7,8]. At the aggregated level, there is a costly revolving door between homelessness and the hospital healthcare system [9].

Viewed through the lens of social determinants, homelessness is both a key driver of poor health and a consequence of adverse social and economic conditions [10]. Whilst access to shelter or housing is a basic social determinant of health and a fundamental human right [11], homelessness itself is socially determined, often the cumulative result of dysfunction in family, social, economic and relational areas [12]. Indeed seminal work by Fitzpatrick and colleagues in Scotland has shown that homelessness is often preceded by life adversities rooted in childhood and other markers of deep social exclusion [13]. The socially determined pathway to homelessness is neither simple nor linear, but often comprises a tangle of factors at the economic, housing interpersonal and individual level [13].

If we are to reduce the impact of homelessness on health, it’s critical to recognise the futility of treating the medical issues associated with homelessness without first acknowledging and then addressing the underlying social issues. The standard medical model does not encompass the realities of being homeless. As articulated by Wise and Phillips [14] in their paper on narratives of healthcare and homelessness:

“Until a health care problem becomes life threatening, a homeless individual will likely choose shelter or food before going to the doctor. These priorities must be considered when dealing with the homeless population. What might, at first, seem like carelessness or noncompliance is, in reality, simply a struggle to survive”.

([14], p. 366)

Adding to these difficulties, the rough sleeping homeless population is notoriously difficult to engage. They have typically experienced high levels of traumatic life experience and negative experiences with health services that do not comprehend or adapt to their specific needs [15]. Homeless individuals often do not seek help until late in the course of their illness, so the opportunity to intervene early in the course of the illness or injury is lost. Medical care is often sought only at an advanced stage of disease when it requires more extensive and expensive treatment.

The many health issues of homeless individuals cluster with, and are exacerbated by, other social determinants of health such as psychological trauma, poverty, unemployment, domestic violence and social disconnection [16,17]. This constellation of underlying social issues challenges traditional clinical boundaries: they are not seen as “medical” problems although they are the major determinants of health for people experiencing homelessness. Awareness and understanding of these underlying issues is critical to effective healthcare but traditional hospital data and records do not necessarily capture this at the aggregate level. In this paper therefore we use a case report format to illustrate the intertwined myriad of social determinants that underlie the frequent hospital presentations of people who are living on the streets.

## 2. Methods

This paper presents three case histories of homeless patients seen by a Homeless Team established in June 2016 within an inner city tertiary hospital in Perth, Western Australia. The Homeless Team is based on the UK Pathway model (pathway.org.uk) involving hospital in-reach by a specialist Homeless Medicine General Practice [18,19]. In Perth, this critical GP in-reach is provided by Homeless Healthcare, a dedicated GP service that has the dual aims of improving the health and welfare of people while they are homeless, and of providing ongoing healthcare and support to enable people to break the cycle of homelessness [20,21]. Staffed by GPs and nurses, Homeless Healthcare provides primary care to people who are homeless in a mix of settings, including drop-in centres for people who are homeless; clinics within shelter and transitional accommodation facilities; a mobile van reaching out to people who are street homeless; as well as via in-reach to the tertiary hospital from which these three case studies are derived (website ref above). Both Homeless Healthcare and the Homeless Team within the tertiary hospital strike to connect people to other services and supports that can break the revolving door between homelessness and health.

The Homeless Team (HT) is part of a wider collaboration with local homelessness community services through the 50 Lives 50 Homes project [22], a multi-agency collaboration using a Housing First model [23] to rehouse and support Perth’s most vulnerable and complex rough sleepers.

The 50 Lives 50 Homes project’s (50 Lives) objective is to assist the most vulnerable of homeless people in Perth to access long-term housing, coupling this with case worker and after hours support to assist patients to secure and sustain housing [24]. It is a cross-sectoral collaborative project between a range of homelessness, health, community support and housing services and includes a unique after hours support service to assist with housing, health and psychosocial issues out of business hours [22,24]. Recognising that homelessness and poor health outcomes are intertwined, a unique feature of the 50 Lives project is that primary healthcare services to 50 Lives patients are provided by Homeless Healthcare GP practice in their 11 practice locations which includes hospital in-reach, multiple community clinics, a street health program and being part of the after-hours support team [24].

### 2.1. Ethics Approval

The three case histories summarised in this paper form part of two concurrent research projects: (i) an evaluation of changes in health service use among patients of Homeless Healthcare with ethics approval from the Human Research Ethics Committee (HREC) of Royal Perth Hospital (RGS0000000075) and The University of Western Australia (UWA) HREC (RA/4/20/4045); and (ii) evaluation of the 50 Lives 50 Homes project that has ethics approval from UWA HREC (RA/4/1/8813), and is also covered by the aforementioned Royal Perth Hospital HREC for access to de-identified data on hospital use for purposes of case studies and analysis of changes in hospital use and associated costs. As these ethics approvals cover the use of de-identified data for the purpose of case studies, individual consent was not required by HREC. In addition, all respondents to the VI-SPDAT survey (from which some contextual information for the case studies has been drawn) have provided signed consent, which includes acknowledgment that findings that do not identify them individually can be “used to inform government, non-government agencies, research and the community about the needs and experiences of people experiencing homelessness” [25].

### 2.2. Data Sources

The case histories draw on a variety of data sources. The healthcare information was obtained from the Perth metropolitan hospital patient database, TOPAS, which is available from all but two hospitals. Clinician observations from patient contacts and consultations were also used. Homelessness data was collected via the VI-SPDAT questionnaire, completed with street present homeless individuals by agencies working with this population. The VI-SPAT is a triage tool, used to identify the vulnerability level and needs of street present individuals. Those who score in the “high acuity” range (score > 9) are eligible for inclusion in the 50 Lives project.

The VI-SPDAT is a validated tool that collects information across the domains of history of housing and homelessness, risks, socialisation and daily functioning and wellness [26]. The Vulnerability Index (VI) was developed to assess key mortality risk factors in people who are chronically homeless (>6 months duration). It was later expanded to include the Service Prioritisation Decision Assistance Tool (SPDAT), assesses the homeless individual’s needs so the appropriate interventions can be identified and prioritised [26]. The VI-SPDAT tool has been widely used in homelessness interventions in the USA, Canada and increasingly, in Australia.

The case histories also include economic estimates of ED visits and inpatient admissions for the three individuals for the period January 2015–September 2017 (33 months). The costs were calculated using the most recent hospital cost data produced by the Independent Hospital Pricing Authority (IHPA) [27]. The IHPA generates a yearly report based on data submitted by Australian public hospitals and is a widely used benchmark for healthcare costs [27]. The cost for admissions to psychiatric units/wards was drawn from the Australian Institute of Health and Welfare’s annual “Expenditure on Mental Health Services” report, also based on 2014–2015 data [28].

Whilst the primary focus of this paper is on the social determinants of health that underlie the over-representation of homeless people in hospital statistics, the case studies also draw on information from the 50 Lives project regarding the status of efforts to connect these three patients to housing and case worker support. As all three patients in the case histories have only been housed recently, it is too early to undertake a full cost effectiveness analysis of changes in hospital use following housing and case worker support. However we include some provisional data on changes in hospital use to illustrate the potential to dramatically improve health and reduce hospital use when housing is provided as a critical first step as part of a social determinants informed response to homelessness and health inequities.

## 3. Case Presentations

### 3.1. Case 1

#### 3.1.1. Background

This 35 years old indigenous man, with no stable accommodation for 10 years, identified the street as where he most frequently sleeps. His highest level of schooling was year 8. His first contact with the adult hospital healthcare system was in 2005 when he was hit by a car at the age of 22 years old, causing severe injuries. By this time, he already had severe ataxia (unsteady gait) and intellectual impairment from heavy solvent and alcohol abuse during his teenage years. In 2012 he had a further major car accident causing multiple leg fractures and by 2013, he was permanently wheelchair bound, homeless and a frequent user of street drugs, solvents and alcohol. He reports being regularly assaulted and having had multiple interactions with police, at the age of 30 years old, he was approved for permanent supported care but always left hospital to return to the streets before this could be found. His combination of permanent physical disability, lack of mobility, brain injury and vulnerability to attack and coercion resulted in him scoring 14 on the VI-SPDAT questionnaire in August 2016, reflecting high vulnerability. When asked ‘what do you need to be safe and well’ he simply answered ‘a house’.

#### 3.1.2. Hospital Presentations and Admissions

Hospital use by this patient for a 27 months period (January 2015 to March 2017) is summarised in Table 1 and the costs of these ED presentations and inpatient admissions have been computed using the sources described in the methods section. His total estimated costs for the 51 ED presentations over the 27 months period January 2015 to March 2017 was $33,456, and the cost of the 28 inpatient admissions amounted to $299,460. In total, this represents a cost to the health system of almost $333,000 or $12,333 per month over a 27 months period.

#### 3.1.3. Current Housing and Health Circumstances

In early April 2017, this patient was found a place in a supported psychiatric care facility. Since then, he has not presented to any hospital or been admitted as an inpatient in the 7 months since being housed. The comparative cost of his past hospital use with the cost of case worker support and housing is discussed at the end of this results section.

### 3.2. Case 2

#### 3.2.1. Background

This 58 years old non-indigenous man had been homeless since his early thirties. He developed schizophrenia in early adulthood and retreated from mainstream life to live under a suburban bridge for the next 26 years, with minimal contact with the medical or psychiatric system. By the age of 55 years old his schizophrenia had “burnt out” but he was experiencing increasingly severe low back pain and leg weakness from degenerative lumbar spine disease. This significantly impacted his mobility and resulted in more frequent ED presentations. By mid-2016, his back pain and leg weakness rendered him unable to mobilise over any significant distance and his presentations to hospitals escalated further. Over the years he had also developed other health problems: severe COPD and hepatic disease due to heavy cigarette and alcohol use respectively and bladder cancer. Despite these issues and very limited mobility he was repeatedly discharged back to live under the bridge after brief hospital admissions. By late 2016, he was wheelchair bound and moved into the Perth Central Business District where homeless services such as food vans were in closer proximity but the risk of assault was considerably greater. He continued to present frequently to hospital asking for assistance with housing and his medical issues, especially his back pain and leg weakness. He scored 14 on the VI-SPDAT questionnaire in August 2016, which reflects high vulnerability.

#### 3.2.2. Hospital Presentations and Admissions

Hospital use by this 58 years old patient for the 29 months period January 2015 to July 2017 is summarised in Table 2 with the associated costs of ED presentations and inpatient admissions. They cost almost $250,000 over the 29 months period; 69 ED presentations costing $45,264 and the 84 hospital bed days adding up to $202,860 (based on IPHA R19 estimates). This does not include other admission costs such as investigations or medications.

#### 3.2.3. Current Housing and Health Circumstances

In July 2016, the hospital Homeless Team connected this patient to the 50 Lives 50 Homes project. He was prioritised for housing due to his high vulnerability and frequent hospital admissions. Through the efforts of his caseworker, he was housed in a friendly aged care hostel facility in early August 2017 and has had no ED presentations since. Regular follow up visits have been made by Homeless Healthcare to him at his new accommodation as part of the After Hours Support Service provided as part of the 50 Lives project. Having lived on the streets for nearly half his life, there have been many adjustments, for example he had spent so long out of mainstream life that he needed instructions to use a TV remote control. Whilst it is early days and premature to compute a cost-benefit analysis in relation to this case study, as discussed in Section 3.4, the unit cost of ED presentations and inpatient admissions far outweighs the costs associated with case worker support and housing.

### 3.3. Case 3

#### 3.3.1. Background

This 31 years old non-indigenous woman spent 18 months homeless after she became estranged from her partner and family due to heavy alcohol use and an eating disorder, both problematic since her late teens. She completed schooling up to year 10 but by the age of 28 she had significant cognitive deficits and brain atrophy due to heavy alcohol use, malnutrition due to the eating disorder and brain injury due to falls and assaults. In late 2016, she became homeless, drinking 2–5 L of wine per day, sleeping on the streets with repeated physical and sexual assaults as well as falls which brought her to hospital EDs on a regular basis. Neither medical nor psychiatric admissions were able to change her dire social circumstances or heavy alcohol intake and this impeded efforts to find her stable accommodation. Her VI-SPDAT score of 14 in late 2016 equates to high vulnerability. When asked in the questionnaire “What do you need to be safe and well?” she responded “stable accommodation and a steady income so I can be financially independent”.

#### 3.3.2. Hospital Presentations and Admissions

Hospital use by this patient over the 27 months period while homeless (January 2015 to March 2017) is summarised in Table 3. Prior to obtaining supported housing in April 2017, this patient had frequent ED presentations and hospital admissions, the basic costs adding up to nearly $623,000, an average of just over $23,000 per month. The eight psychiatric admissions had an average LOS of 26.7 days and accounted for 37% of total bed days where the 44 medical (non-psychiatric) admissions were much shorter with an average LOS 2.3 days with most being overnight stays, generally for alcohol intoxication +/− injury.

#### 3.3.3. Current Housing and Health Circumstances

In late March 2017, this patient was housed at a mixed gender supported accommodation facility but required considerable additional psychiatric and general support due to ongoing heavy drinking. Homeless Healthcare and the 50 lives After Hours Support Service have seen her frequently as a result. There was steady improvement for a few months, but then she was unfortunately the victim of an assault, resulting in further ED presentations and increased drinking. Nevertheless, in the 6 months since being housed, she has had only 5 ED presentations and no admissions. An alcohol rehabilitation admission is now planned.

### 3.4. Potential for Cost Savings Through Housing and Support for These Patients

#### Potential for Cost Savings through Housing and Support

As it is early days since these three patients commenced receiving case work support and were housed, it is not possible to compute a cost-benefit analysis comparing hospital costs before and after receiving support with the costs of case worker support and accommodation. However, Table 4 shows indicative unit cost estimates for case worker support and several options for supported accommodation that would need to be taken into account. The estimated cost for supported accommodation for people who have been homeless is based on the work of Zaretzky and Flatau (2013), which reports cost/patient/day for supported accommodation services equivalent to $82/support day, $49 recurrent cost and $33 opportunity cost of capital (inflation adjusted to $2016) [29]. This has been converted to a cost/week value in Table 4. The cost of case worker support is based on the average award wage salary for case workers in homelessness sector, and apportioned based on average caseload of 10 patients. Several accommodation options have been included, ranging from a public housing rental, supported accommodation in a homelessness service, through to residential mental health or aged care facilities.

## 4. Discussion

An accumulation of evidence from around the world shows a strong association between homelessness and health disadvantage [9]. The preceding three case histories illustrate the tangled webs of social determinants that underlie this relationship. They present a small sample of the common and complex problems found in the lives of people experiencing homelessness.

In the homeless population, the social determinants of health often start with adverse early life experiences and trauma, followed by poor educational outcomes and disengagement, involvement in drugs, unstable relationships, erratic work history and often imprisonment [33]. It is the compounding effect of these multiple adverse life experiences and consequent lack of a stable support network which leads to homelessness [13,33]. The relationship between homelessness and health is bi-directional and compounding because housing and health strongly influence each other [34,35]. Just as untreated mental illness can precipitate homelessness, homelessness is itself a significant risk factor for poor mental health [22,23].

The intertwined nature of social disadvantage and health has major implications for health policy and practice. The three case histories illustrate the extreme end of a wider phenomenon: health issues being managed within an entirely medical paradigm, without concurrent management of strong underlying social determinants.

We discuss three important lessons to be drawn from these stories:

Firstly, in order to reduce the enormous health inequalities seen in the homeless population, we need to view homelessness (and other types of severe social disadvantage) as a combined medical and social issue. Addressing homelessness is, itself, an important form of healthcare, not a separate “non-health” issue. The dismal results of the traditional silo approach and narrow medical notion of health, are amply illustrated by the three case histories. Indeed the futility of treating homeless patients and sending them back to the social conditions that make them sick has been powerfully questioned by Marmot in his recent book The Health Gap [36].

An example of how to better address the problem is the Pathway program in the UK [19]. It brings community service organisations and homeless medicine GPs into hospitals, to the bedside, to work alongside the hospital team. This provides combined medical and social interventions both within the hospital and beyond. The desired outcome is durable social stabilisation and engagement with long-term primary care, which results in dramatic falls in hospital healthcare utilisation and markedly improved patient health and wellbeing, as our case histories illustrate. The Pathway model is highly transferable if there are Homeless Medicine practitioners working in the community. In our Perth inner city public hospital, this expertise is provided by GPs and nurses from the Homeless Healthcare practice [21] who do hospital in reach as well as providing ongoing primary healthcare in the community. This continuity of care from hospital to general practice facilitates long term engagement from homelessness to re-housing. As life stabilises for the individual via housing, the myriad of issues around mental health, physical health and substance abuse can be addressed.

Secondly, our society and the public purse currently spend an extraordinary amount of money on hospital healthcare for people experiencing homelessness. The expenditure undoubtedly stops or postpones many homeless individuals dying from the severe injuries and illness which result from their poor social situation. However, as the case histories illustrate, the traditional medical model does not improve overall health nor reduce healthcare usage over time. The cumulative healthcare costs continue to accrue and often accelerate as health and social issues worsen, amply illustrated by Case 2.

The huge economic cost of homelessness was brought to wider public attention in 2006 through the story of “Million Dollar Murray”, a chronically homeless alcoholic man living on the streets of Reno in Nevada [37]. Murray Barr’s heavy drinking brought him into astonishingly frequent contact with the police and hospital services, at a cost which vastly exceeded that of simply paying to house and support him [37]. The local authorities concluded that over 10 years “it cost us one million dollars not to do something about Murray” [37]. This economic rationalism has changed the justification for providing housing and support away from one based entirely around humanitarian considerations. Recognizing that the economic cost of homelessness outstrips that of providing housing and support is producing shifts in public policy decisions round homelessness. There is a move away from the customary but poorly effective “Housing Ready” approach in which homeless individuals must engage with multiple services and programs over months to years and become stable before they can be housed. It is being replaced by the “Housing First” model which provides stable housing as the first, and not last, step. Housing First programs have shown high rates of achieving and retaining housing and reduced health service use in the US [38,39,40], Canada [41,42,43] and Australia [44]. One study conducted in America illustrated a retention rate of 84% two years after program inception [45], with such stability of housing allowing for health outcomes and their underlying determinants to be more effectively addressed. In the 50 Lives 50 Homes project, which has housed 71 individuals and families to date to date, there has been a 90% retention rate among the 28 individuals housed for one year or longer [22].

In this new era globally of economic pragmatism, funding Housing First rehousing and support programs should be considered as cost cutting measures rather than expenditure. The potential savings multiply when the financial impact of homelessness is examined across the many government services used heavily by homeless people. In this article we examined only Health costs but there are major financial impacts in the areas of Justice, Corrections, Ambulance Services, Child Protection and Family Services and Social Security. An evidence synthesis recently published in The Lancet on the effectiveness of interventions for marginalised and excluded populations, including people who are homeless, concluded that housing first response to homelessness can improve a range of health and social outcomes, particularly among those with mental health or substance use co-morbidities [46].

Moreover when economic rationalism drives decisions around homelessness policy, it strips them of moral considerations of whether homeless individuals are “deserving” of publically funded assistance. Paradoxically, applying pure economic rationalism to homelessness policy renders the decisions more just and humane. Thus whilst there is a ‘cost saving’ case that can be made for both addressing the underlying determinants of homelessness, this should never overshadow the fundamental right of all human beings to health, dignity and a place to call home. As articulated recently by Luchenski and colleagues in The Lancet [46], government and social policy needs to recognize that the vast health inequalities seen in people who are homeless and other marginalized population groups, have their root causes in structural systemic disadvantages; there is therefore a social justice imperative to reverse some of the exclusionary processes that have led to this, and a need to prioritise he material and the health needs of those for whom greatest inequities exist [46].

Thirdly, the case study approach taken in this paper was intentional, revealing the human reality behind the homelessness statistics. The life stories of these three previously homeless individuals are rich in lessons, not only for professionals who work with the homeless population in healthcare and other settings but also those who make policy or financial decisions that affect them. Whilst as individual cases these are not generalizable, the prevailing social determinants and the high rates of hospitalisation are not at all atypical for this population group. There is a growing body of empirical data documenting the stark inequities in the health of people who are homeless, but individuals’ stories can go beyond aggregated numbers, to show the relentless accumulation of social disadvantage and adversity which leads to homelessness, the stark realities of living on the street and how difficult it can be to find a path back to mainstream life. The storytelling approach was recently used by Australian television channel, SBS, in their widely viewed 2017 “Filthy Rich and Homeless” television series [47]. Such stories show us the human face of homelessness and challenges us to consider how our society disregards their need for the most basic elements of life which most of us take for granted.

The voices and lives of homeless individuals are rarely seen in health and medical literature [14] or [48] as case reports. A recent Canadian paper for example found only a handful of examples where homelessness was included in case studies used in medical school teaching, and those that did lacked information about the individual’s social context [48]. Given the over-representation of people experiencing homelessness in healthcare settings such as EDs, hospital wards and mental health units, it is imperative that health professionals and health systems understand the interplay between health and social issues.

### Limitations

This paper has intentionally taken a case study approach to provide richer contextual elucidation of the way in which socially determined factors contribute to and exacerbate the vast health inequities that exist among people who are homeless. While real-world insights such as case histories are valuable, they clearly do not encompass the varied backgrounds and circumstances of the whole homeless population. The generalisability of our observations and findings are therefore limited. Nonetheless we contend that the types of social determinants that manifest in the lives of people who are homeless are by no means atypical, and this is supported in the published literature. The three case histories were chosen because they were high users of hospital healthcare who had been seen by our Homeless Team and were part of the 50 Lives 50 Homes program, Perth’s Housing First program described above. Individuals experiencing homelessness are over represented at our inner city hospital, comprising 65–70% of the ED’s 20 most frequent presenters.

The economic costings and discussion in this paper do not purport to constitute a comprehensive cost benefit analysis, as this is beyond the scope of a case report paper, and a longer period of data post housing and support is needed to be comparable to the health data time period. Our main intent was to capture the enormous health system burden that can recur for several years (at least) when people exit hospitals back into homelessness. Hence we considered it relevant to present a longer period of health data (27 months for cases 1 and 3, 33 months for case 2) to convey this. As we wanted to present three very current scenarios that hospitals with high attendances by people who are homeless might face, the time period following housing and support is much shorter and therefore not directly comparable. However, the fact that hospital usage has dropped in all three cases within even a short time period is important to convey, as it shows how ‘housing first’ combined with individual case worker support and follow up primary healthcare can serve as a powerful circuit breaker to the revolving door between homelessness and health.

The costings for hospitalisation costs, housing and case worker support are based on publically available data. We acknowledge that these costs are based on reported ‘average costs’ and there is will be variability at the individual case level. Nonetheless, it has been argued elsewhere that if anything, average costs for ED presentations and inpatient admissions are in fact likely to under-estimate the costs borne by the health system for homeless patients who have greater co-morbidities and unaddressed health needs [49].

## 5. Conclusions

Housing has long been recognised as a fundamental human right and a core social determinant of health [50]. This is often forgotten however at the coalface of medical care and social policy decision making, particularly in this era of overburdened health systems, and pressures to respond to the most immediate acute health needs when resources are constrained.

Taking more pragmatic and informed approaches and using evidence-based interventions such as Housing First and integrated medical and social care for homeless patients not only improve lives but also reduces ineffective, futile public service spending. Ending homelessness requires not only a vigorous response to existing homelessness but upstream intervention around a raft of early social determinants. This reaches right back to the roots of homelessness that generally start in childhood: tackling the poverty, violence, trauma, educational disadvantage and discrimination which underpin homelessness in our society.

## Figures and Tables

**Table 1:** Case 1: Hospital presentations and admissions (January 2015–March 2017).

Year	ED Presentations (n) ^	Hospital Admits (n)	Hospital LOS (Days) ^^	Estimated Cost
2015	36	17	53	$151,611
2016	7	5	52	$130,172
January–March 2017	8	6	19	$51,133
Total	51	28	124	$332,916

^ $656 per presentation; ^^ $2415 per day.

**Table 2:** Case 2: Hospital presentations and admissions (January 2015–July 2017).

Year	ED Presentations (n) ^	Hospital Admits (n)	Hospital LOS (Days) ^^	Estimated Cost
2015	13	7	17	$49,583
2016	28	21	39	$112,553
January–July 2017	28	11	28	$85,988
Total	69	39	84	$248,124

^ $656 per presentation; ^^ $2415 per day.

**Table 3:** Case 3: Hospital presentations and admissions (January 2015–March 2017).

Year	ED Presentations (n) ^	Hospital Admits (n)	Hospital LOS (Days) ^^	Psychiatric Admits (n)	Psychiatric LOS (Days) ^^^	Estimated Cost
2015	46	18	48	4	135	$325,916
2016	35	17	30	2	68	$185,986
January–March 2017	22	6	40	2	11	$125,684
Total	103	41	118	8	214	$637,586

^ $656 per presentation; ^^ $2,415 per day; ^^^ $1,332 per day.

**Table 4:** Accommodation and case worker cost estimates.

Accommodation Options	Cost/Day	Cost/Week
Support accommodation (homelessness services) *	$82	
Public housing average recurrent cost/dwelling/week **		$195
Residential mental health service ***		
24 h staffing	$522	
Non 24 h staffing	$181	
Case worker support		
Case worker/patient/week ****		$115
Comparative cost of hospital use		
ED presentation *****	$656	
Inpatient admission *****	$2415	

**Sources:** * SHS cost based on Zaretzky and Flatau 2013 [29]. ** Average recurrent cost/public housing dwelling ($10,122/year) Zaretzky and Flatau 2015 [30]. *** The average estimated cost for a person in a residential mental health service in WA (2014–2015, most recently available data) was $522 with 24 h staffing, and $181 without 24 h staffing [31]. **** Based on July 2017 level 5 pay point 1 award for a community services worker with 30% on-costs and typical caseload of 10 patients [32]; ***** Based on IHPA Round 19 National Hospital Cost Data [27].
